# Administration of Nrf-2-Modified Hair-Follicle MSCs Ameliorates DSS-Induced Ulcerative Colitis in Rats

**DOI:** 10.1155/2021/9930187

**Published:** 2021-10-27

**Authors:** Lin Zhou, Fengjuan Yan, Rui Jiang, Jing Liu, Limin Cai, Yongchen Wang

**Affiliations:** ^1^Department of General Practice, The Second Affiliated Hospital of Harbin Medical University, Harbin, Heilongjiang 150086, China; ^2^Department of Dermatology, The First Affiliated Hospital of Harbin Medical University, Harbin, Heilongjiang 150001, China

## Abstract

Ulcerative colitis (UC) is a common chronic nonspecific intestinal inflammation of unknown etiology associated with a low cure rate and a high relapse rate. Hair follicle mesenchymal stem cells (HF-MSCs) are a class of pluripotent stem cells that have differentiation potential and strong proliferation ability. Nuclear factor red system related factor (*Nrf-2*) is a key factor in the oxidative stress response. Dextran sulfate sodium- (DSS-) induced rat UC models closely mimic human UC in terms of symptoms and histological changes. Animals were divided into five groups, including a healthy group and UC model rats treated with normal saline, *Nrf-2*, HF-MSCs, or *Nrf-2*-expressing HF-MSC group. Based on the expression of intestinal stem cells, inflammatory factors, anti-inflammatory factors, and disease activity index scores, *Nrf-2*-expressing HF-MSCs had the most obvious therapeutic effect under the same treatment regimen. This study provided a new potential clinical treatment option for ulcerative colitis.

## 1. Introduction

Inflammatory bowel disease (IBD) is an idiopathic intestinal inflammatory disease which involves the colon and rectum as well as the ileum. The main clinical symptoms of the disease are enteritis, diarrhea, and intestinal bleeding, and the disease can be debilitating. The two core kinds of IBD include ulcerative colitis (UC) and the disease of Crohn [1–3]. The etiology of UC remains unclear, but it is generally believed to be related to genetic susceptibility, microbial infection, and environmental invasion [4]. Various drugs, such as tacrolimus, corticosteroids, and azathioprine are used to treat UC. These medications can alleviate UC symptoms; however, the adverse effects limit their therapeutic use [5–7]. Therefore, new treatment options are warranted to improve the clinical management of UC patients.

Mesenchymal stem cells (MSCs) exhibit tissue migration, multidirectional differentiation, self-renewal, and anti-inflammatory properties and are new therapeutic candidate for IBD [8–10]. Hair follicle mesenchymal stem cells (HF-MSCs) offer notable advantages, such as ease of collection, lack of age and sex limitation, and long-term survival in the human body owing to their low immunogenicity. In addition, autologous transplantation alleviates immune rejection and ethical issues; moreover, HF-MSC can perform tissue repair [11, 12]. Therefore, HF-MSCs are deemed an ideal cell source for cell engineering.

*Nrf-2* has been considered to be a transcription factor that can bind to the antioxidant response element and exert anti-inflammatory responses, detoxification, and radiation protection in the human body [13]. *Nrf-2* activates antioxidant enzymes to remove excess oxidative free radicals, which are harmful to DNA, cells, and tissues. Further, *Nrf-2* accelerates wound repair and tissue remodeling. Its protective effects in the colon have also been validated; intestinal integrity is protected by *Nrf-2* via inducing phase II detoxifying enzymes and regulating proinflammatory cytokines [14].

This study determined whether *Nrf-2* overexpressing HF-MSCs provide better therapeutic impacts within UC which is induced by dextran sulfate sodium (DSS), compared to the nonengineered HF-MSCs. We examined the localization of HF-MSCs overexpressing Nrf-2 within rats' intestinal tissue and made a comparison of the therapeutic impacts with four other experimental groups. Therefore, engineering *Nrf-2* into HF-MSCs may yield favorable results in treating UC.

## 2. Methods and Materials

### 2.1. Animal Experimental Design

Male rates of SD were bought from the Animal Experimental Center of the Second Affiliated Hospital of Harbin Medical University (Harbin, China). One-week-old SD rats were used to obtain HF-MSCs. Ten rats were used to determine whether DSS modeling was successful. The rest of the rats had been classified randomly into 5 groups (*n* = 5/group): a healthy group which did not receive any treatment, a saline group that was intravenously administered a 0.9% NaCl solution, an *Nrf-2* group that was administered *Nrf-2* adenovirus directly, an HF-MSC group that received HF-MSCs (1.0 × 10^6^ cells/rat), and an *Nrf-2-*HF-MSC group that was intravenously administered Nrf-2-overexpressing HF-MSCs (1.0 × 10^6^ cells/rat). The experimental protocol was approved by the Animal Care and Use Committee of Harbin Medical University (SYDW2018-132).

### 2.2. Cell Isolation, Culture, and Identification

HF-MSCs were isolated from one-week-old male SD rats. After anesthetizing the rats, the skin surrounding the beard was cut off. 0.1% type IV collagenase was used to incubate the tissue for 2 hours. The hair follicles were separated from the connective tissue sheath under a stereomicroscope and seeded in a 24-well plate at one hair follicle per well. The medium (DMEM/F12 containing 10% FBS, 100000 U/L penicillin, pH = 7.4) was renewed daily. When cells grew to 70-80% confluence, they were passaged. HF-MSCs of the third generation were cultured within an atmosphere with 5% CO_2_ at 37°C, and the adipogenic and osteogenic differentiation medium had been changed. The adipogenic and osteogenesis differentiation had been verified by Oil Red O (Sigma-Aldrich Chemical Company, St Louis, MO, USA) staining and Alizarin Red (Sigma-Aldrich Chemical Company, St Louis, MO, USA) staining after being incubated for 14 days, respectively. HF-MSC markers were detected by FACS. Approximately 1 × 10^6^ cells were incubated with 1.0 *μ*L of monoclonal antibodies against CD44, CD90, CD45, and CD31 (BD Biosciences, Mountain View, CA, USA). An isotype control antibody was used as a negative control. Cells were explored by flow cytometry (BD, San Jose, CA, America). The fluorescence signal was evaluated using Cell Quest software.

### 2.3. Cell Transfection

To construct a lentiviral *Nrf-2* overexpression plasmid vector, we designed a specific PCR primer pair to simultaneously introduce the enzyme digestion site. The *Nrf-2* gene was ligated into the T vector (HANBIO, Shanghai, China), and the target gene region was cut from the T vector and transferred into a lentiviral overexpression vector. The final plasmid was transfected into 293T cells, and the medium was changed 6 h after transfection. After 24 and 48 hours of culture, cell supernatants rich in lentiviral particles were collected, and the virus supernatants were concentrated by ultracentrifugation. The cells had been seeded at 1 × 10^5^ cells/well into 24-well plates at 18–24 h before lentiviral transfection. The number of cells during lentiviral transfection was approximately 2 × 10^5^ cells/well. The next day, the medium was replaced by using 2 mL of fresh medium which contained 6 *μ*g/mL polybrene, and a proper amount of virus suspension was added. The culture was continued for 24 hours, and the medium-containing virus was replaced by using fresh medium. To confirm successful transfection, *Nrf-2* protein expression was verified by western blotting.

### 2.4. Enteritis Model Establishment and Evaluation

DSS was used to establish the rat UC model [15]. In brief, 5% DSS (Shanghai Yuanye Bio-Technology, Shanghai, China) 3.5 mL/100 g daily was administered to rats by gavage. DAI, body weight, stool consistency, and occult blood were monitored to evaluate DSS-induced UC [16, 17]. Weight loss was scored as 0, 1, 2, 3, and 4 for no, 1–5%, 6–10%, 11–15%, and >15% weight loss, respectively. To evaluate fecal viscosity, well-formed feces was scored 0, semiformed paste feces glued to the anus was scored 2, and watery diarrhea was scored 4. Hemorrhage negativity was scored 0, hemorrhage positivity was scored 2, and severe hemorrhage was scored 4. The DAI computed the total of the scores divided by 3. UC rats (DSS gavage 2 weeks) and healthy rats (saline gavage 2 weeks) were sacrificed under ketamine (60 mg/kg) and xylazine (10 mg/kg) anesthesia, and the intestine was removed. Intestinal length was compared between the two groups; ulcer sites in the UC group were stained with Evan Blue. Colon tissue sections were stained with hematoxylin, differentiated with 1% ethanol hydrochloride, stained with 1% water-soluble eosin, dehydrated with anhydrous ethanol, air-sealed, fixed, and later observed through a microscope (Olympus, Tokyo, Japan). Histological scoring is based on the following criteria: epithelial injury degree: 0—normal morphology, 1—crypt loss, 2—big region goblet cell loss, 3—goblet cell loss, and 4—large area crypt loss; infiltration degree of inflammatory cells: 0—infiltration of inflammatory cells to the mucosal muscle layer, 1—extensive infiltration of inflammatory cells and thickening of the edema of the mucosal layer, 2—noninflammatory cell infiltration, 3—infiltration of inflammatory cells only around the crypt, and 4—inflammatory cells infiltrating the submucosa. The sum of the two scores is the histopathology score [18].

### 2.5. Immunofluorescence Staining

The colon, liver, spleen, kidneys, and lungs were fixed with paraformaldehyde and incubated with sucrose. Frozen tissues were cut into 5 *μ*m frozen parts, being blocked for 20 minutes at normal temperature by using normal goat serum. Later on, these parts were incubated by using anti-Cdx2 (1 : 200, Santa Cruz Biotechnology, Santa Cruz, CA, USA), anti-PCNA (1 : 200, Abcam Cambridge, MA, USA), anti-TERT (1 : 200, Santa Cruz Biotechnology, Santa Cruz, CA, USA), and anti-Musashi-1 (1 : 200, Santa Cruz Biotechnology, Santa Cruz, CA, USA) antibodies a night at 4°C, followed by goat anti-rabbit IgG-Cy3 (Alexa Fluor 594) or incubation with donkey anti-sheep IgG (H+L) in a wet box for an hour at 37°C. Nuclei had been stained by using DAPI. Then, the sections were fixed, blocked, and observed under a laser confocal microscope. Semiquantitative analyses were implemented with ImageJ (National Institutes of Health, Bethesda, America).

### 2.6. Immunohistochemical Staining

Intestinal samples were embedded in paraffin blocks, sliced, dewaxed in xylene, and hydrated with ethanol. The sections were immersed in a 3% hydrogen peroxide solution for 20 minutes to block endogenous peroxidase activity. Then, they were incubated with antibodies against Cdx2 (1 : 200), PCNA (1 : 250), TERT (1 : 100), and Musashi-1 (1 : 200) for a night at 4°C, followed by incubation with SignalStain® Boost IHC detection reagent (horseradish peroxidase- (HRP-) conjugated antibodies; Cell Signaling Technology, Trask Lane Danvers, MA, USA) and diaminobenzidine staining. After the sections were sealed, protein expression was observed under a microscope, and semiquantitative analysis was performed using ImageJ.

### 2.7. Western Blotting

Intestinal samples were lysed using appropriate amounts of lysis buffer. The lysates were centrifuged, and the protein concentrations in the supernatants were measured. The same amount of protein was electrophoresed under constant voltage and transferred into a PVDF membrane. The membrane was incubated with primary antibodies to *β*-actin (1 : 1000, Santa Cruz Biotechnology, Santa Cruz, CA, USA), PCNA (1 : 250), Musashi-1 (1 : 200), TERT (1 : 100), and Cdx2 (1 : 400) at 4°C overnight and later with HRP-labeled antibodies (1 : 20,000; Bio-Rad Laboratories, America) at 37°C for 45 minutes. Protein bands had been visualized by adopting electrochemiluminescence (ECL Plus Western Blotting Detection Kit). Finally, band densities had been measured by a gel image processing system (Gel-Pro Analyzer).

### 2.8. Total RNA Extraction and RT-qPCR

mRNA levels of *IL-10*, *IL-13*, *IL-1β*, and *TNF-α* were measured by RT-qPCR. The whole RNA had been extracted from frozen tissues by adopting a TRIzol extraction kit (Invitrogen, Carlsbad, CA, USA) in accordance with the protocol of the manufacturer. The RNA concentration in each sample was determined by UV spectrophotometry (NanoDrop 2000). The RNA had been reverse-transcribed into cDNA by adopting a commercial kit (Bioneer, Korea) per the instructions of the manufacturer. PCRs were conducted using the following primers: TNF-*α* F: CGGAAAGCATGATCCGAGAT, R: AGACAGAAGAGCGTGGTGGC; IL-1*β* F: TTCAAATCTCACAGCAGCAT, R: CACGGGCAAGACATAGGTAG; IL-13 F: TATGGAGCGTGGACCTGACA, R: AACTGGGCTACTTCGATTTT; IL-10 F: CCAGTCAGCCAGACCCACAT, R: GCATCACTTCTACCAGGTAAAAC; and *β*-actin F: GGAGATTACTGCCCTGGCTCCTAGC, R: GGCCGGACTCATCGTACTCCTGCTT. Reaction conditions (two-step method) are shown as follows: 40 cycles of denaturation for 15 seconds at 95°C, predenaturation for 10 minutes at 95°C, and 60 seconds at 58°C. Quantitative fluorescence analysis was performed using the Exicycler™ 96 Fluorometer (Bioneer, Korea), and the data were analyzed using the 2^−*ΔΔ*^ CT method.

### 2.9. Enzyme-Linked Immunosorbent Assay (ELISA)

The colon tissues collected from each group were frozen. The tissues were homogenized with cold physiological saline to make 1% tissue homogenate and centrifuged at 14000 rpm for 15 minutes at 4°C, and the supernatant had been gathered. ELISA had been implemented following the instructions of the manufacturer, to determine the superoxide dismutase (SOD), lipid hydroperoxide (LPO), and malondialdehyde (MDA) content.

### 2.10. Statistical Analysis

The resources have been reported to be the mean ± standard deviation (SD). Every statistical analysis had been implemented by adopting SPSS Statistics 23.0 (Chicago, USA). Normality had been evaluated by adopting the Shapiro-Wilk examination. A comparison among these groups had been implemented by adopting one-way ANOVA or the Kruskal-Wallis nonparametric examination. The tissue biomarkers' correlation among each other and with the histological scores had been evaluated by adopting Spearman rank correlation. A two-sided *P* value ≤ 0.05 had been regarded to be greatly important. Every figure was ready by adopting GraphPad Prism 5.0 and Photoshop 7.1.

## 3. Results

### 3.1. Cell Isolation and Characterization

**Figure 1:**
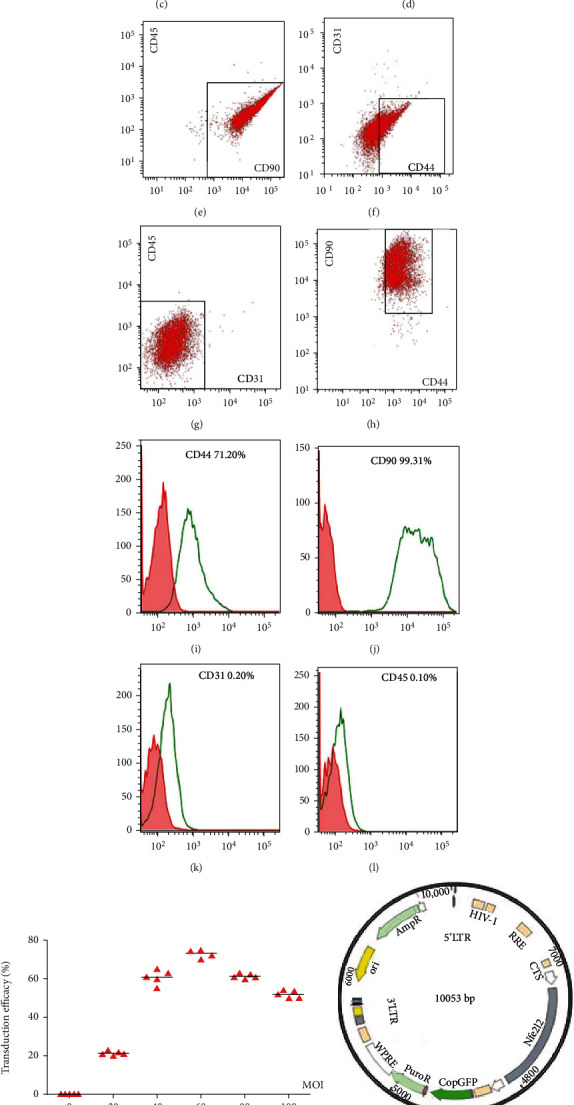
Characteristics of HF-MSCs and establishment of *Nrf-2*-modified HF-MSCs. (a) After 72 h of culture, numerous HF-MSCs aggregated near the hair follicles (20x). (b) Third-generation HF-MSCs (20x). (c, d) Osteogenic and adipogenic differentiation of HF-MSCs. (e–l) Detection of CD44, CD90, CD45, and CD31 by flow cytometry. (m) Relationship between viral transduction efficiency and multiplicity of infection (MOI). Data are expressed as the mean ± SD. (n) Construction of the lentiviral *Nrf-2* overexpression plasmid. (o) GFP-labeled *Nrf-2* was transfected into DAPI-stained HF-MSCs, and obvious blue and green fluorescence was visible under the microscope (20x). (p) Western blot analysis of HF-MSCs transduced with *Nrf-2*-harboring virus construct.

Morphologically, *in vitro*-cultured HF-MSCs isolated from male SD rats were small in size, with a large nuclear/plasma ratio. Cells grew adherently in the shape of spindles or irregular triangles on the culture plate surface. They had an oval nucleus and cytoplasmic protrusions of 2–3 cm (Figures 1(a) and 1(b)) [19]. HF-MSCs have the ability not only to regenerate but also to differentiate into bone, cartilage, and adipose tissue (Figures 1(c) and 1(d)). Maintenance of the HF-MSC antigen phenotype was assessed by fluorescence-activated cell sorting (FACS). The outcomes indicated that CD44 was expressed by 71.20% of cells, and more than 99.31% of cells expressed CD90. On the contrary, CD31 was expressed by no more than 0.20% of cultured cells, and 0.10% expressed CD45 (Figures 1(e)–1(l)). To generate HF-MSCs overexpressing *Nrf-2*, an *Nrf-2* lentiviral vector was constructed (Figure 1(n)). An optimal multiplicity of infection (MOI) (i.e., the ratio of virus to the number of cells when infecting) was determined (Figure 1(m)). Healthy HF-MSCs of passage three were seeded into a 24-well plate and transfected at the optimal MOI. Green fluorescent protein- (GFP-) labeled *Nrf-2* expression was monitored for 48 h after transfection (Figure 1(o)), and *Nrf-2* protein expression was confirmed by western blotting (Figure 1(p)). On the seventh day after *Nrf-2* transfection, there was no change in the appearance of the HF-MSCs, as revealed by bright-field microscopy.

### 3.2. Animal Model Establishment and Evaluation

**Figure 2:**
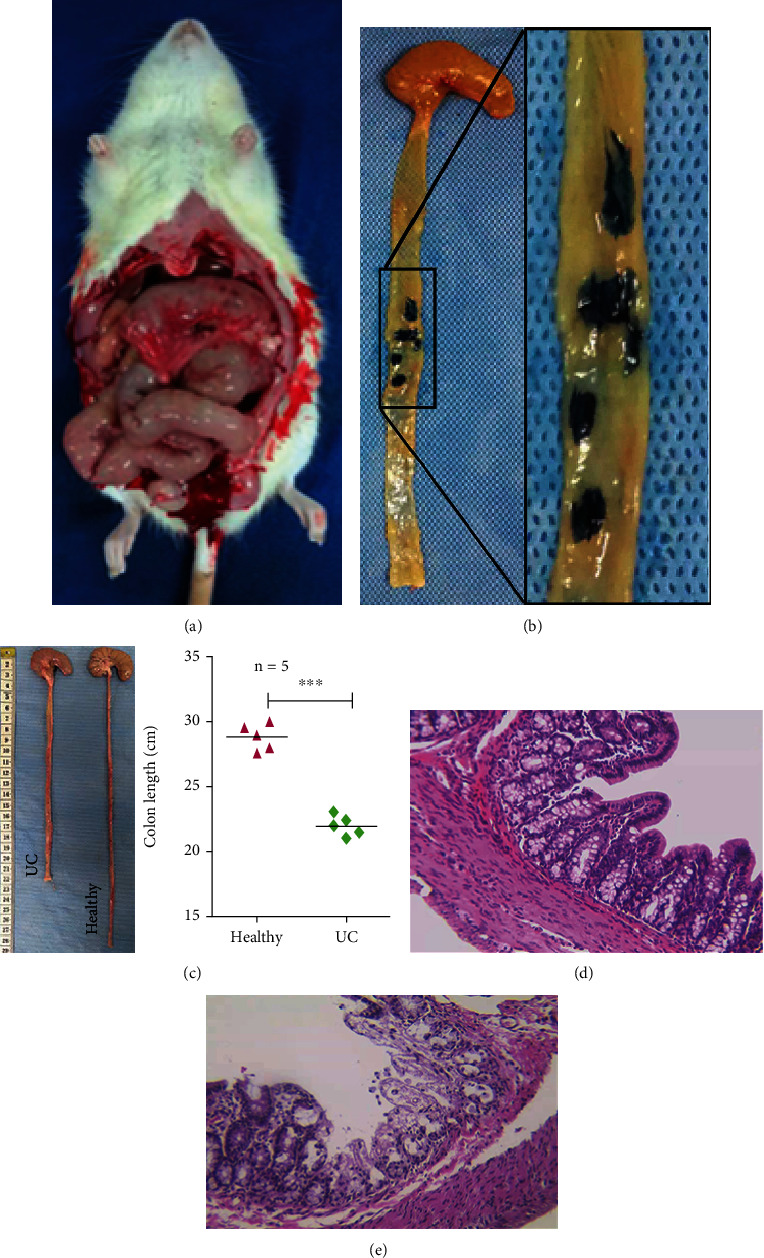
Establishment of the Rat Ulcerative Colitis Model. Photographs of the abdominal cavity of rats after 5% DSS gavage for 14 days. (b) Evans blue staining of the intestine of UC rats. (c) Intestinal length of healthy rats and UC rats. Data are expressed as the mean ± SD (*n* = 5, ^∗∗∗^*P* < 0.001). (d) Comparative HE staining between healthy rats and UC rats.

Based on a comparison of DSS gavage with DSS administration via the drinking water to establish the UC model, we chose the former method because it was more effective and economical. On the 7th day after DSS gavage, the rats began to develop symptoms, including bloody diarrhea, weight loss, trembling, and laziness. On the 14th day, the disease activity index (DAI) score indicated the presence of UC, suggesting successful model establishment. The rats were sacrificed, and hyperemia and edema of the colon were observed (Figure 2(a)). Staining of colon tissues with Evans Blue revealed inactivation of cells in the ulcer site (Figure 2(b)). The colon length in UC rats and healthy rats was measured using a ruler. Colon length had been greatly higher in healthy rats than in UC rats (*P* < 0.05) (Figure 2(c)). Histological examination of the rats based on hematoxylin-eosin staining showed that in the model rats, goblet cells were lost, crypts had irregular shapes, the normal colonic mucosal structure had disappeared, and large numbers of inflammatory cells were present (Figures 2(d) and 2(e)).

### 3.3. Effects of the Different Treatments in the Animal Model

**Figure 3:**
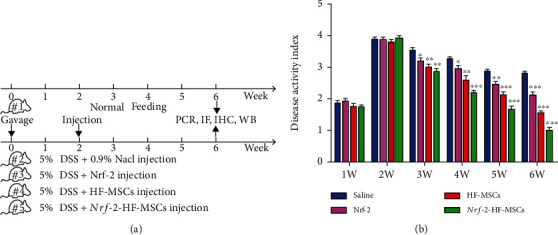
Effect of *Nrf-2* expression and HF-MSCs on ulcerative colitis. (a) Overview of the experimental groups and the timeline in this study. (b) Changes in the DAI in rats of each group over time. Data are expressed as the mean ± SD (*n* = 5; ^∗^*P* < 0.05, ^∗∗^*P* < 0.005, and ^∗∗∗^*P* < 0.001 vs. saline group).

In the second week of the experiment (when the modeling was successful), the animals were divided into five groups, including one healthy control group and four groups of UC model rats treated with 0.9% NaCl, *Nrf-2*, HF-MSCs, or *Nrf-2-*HF-MSCs, which were injected via the tail vein three times a week for four weeks (Figure 3(a)). DAI scores for each treatment group of rats were recorded (*P* < 0.05) (Figure 3(b)). The DAI score in the saline-treated group decreased slightly, probably due to the self-healing capacity of the rats. The mean score of the *Nrf-2* group was lower than that of the normal saline group, whereas the score of the HF-MSC group was lower than that of *Nrf-2*, indicating that HF-MSCs were more effective in treating UC in rats than *Nrf-2*. From the beginning of transplantation to the end of the experiment, the HF-MSC+*Nrf-2* group had the lowest mean DAI score compared to the other groups. In the fourth week of treatment, rats in the HF-MSC+*Nrf-2* group exhibited mild symptoms.

### 3.4. Investigation of the Mechanism Underlying the Treatment Effects

**Figure 4:**
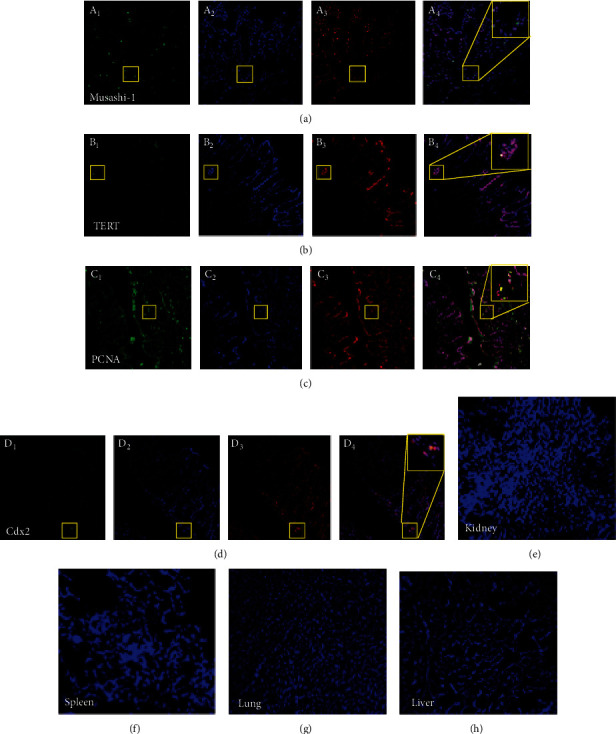
Colonization and proliferation of *Nrf-2*-HF-MSCs in the injured intestine. (a–d) PKH26-labeled cells (red) colocalized with Musashi-1, TERT, PCNA, and Cdx2 (green) in the intestine epithelium after *Nrf-2*-HF-MSC transplantation. The cell nuclei are labeled with DAPI (blue). Scale bar, 50 *μ*m. (e–h) Few positive cells were found in other organs (liver, spleen, kidneys, and lungs).

In the 6th week, all mice were sacrificed, and colon tissues of the rats in each group were analyzed by immunofluorescence (IF), immunohistochemistry (IHC), western blotting (WB), and RT-qPCR experiments. In IF, IHC, and WB experiments, four intestinal stem cell (ISC) markers, including Musashi-1 RNA-binding protein, telomerase reverse transcriptase (TERT), caudal-type homeobox transcription factor 2 (Cdx2), and proliferating cell nuclear antigen (PCNA), were analyzed. IF results showed that HF-MSCs+Nrf-2 labeled with green fluorescence protein were observed in the injured intestine, but not in healthy tissues (including the kidneys, spleen, lung, and liver) (Figures 4(a)–4(h)). The results indicated that the HF-MSCs migrated to the damaged intestinal tissue and exerted therapeutic effects in UC. The homing of HF-MSCs occurs in two continuous processes, namely, mobilization and migration. Adhesion factors, chemokines, cytokines, growth factors, and the extracellular matrix are also involved in the process.

**Figure 5:**
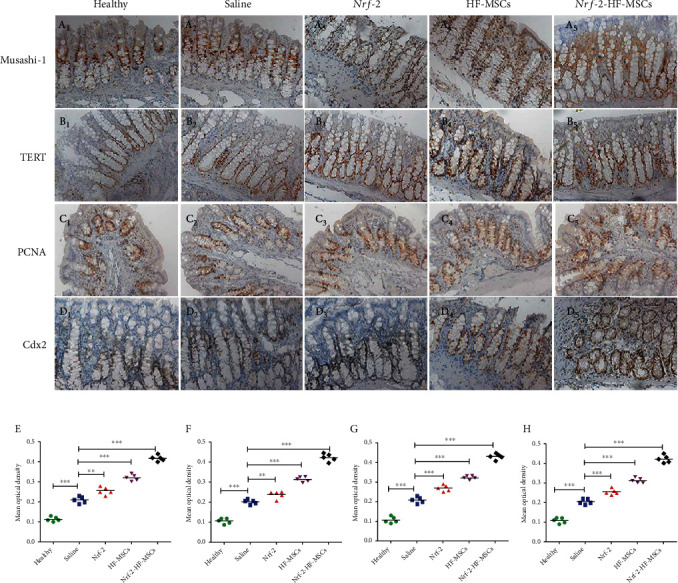
*Nrf-2*-HF-MSC proliferate in the injured intestine. (a–d) Immunohistochemical detection of Musashi-1, TERT, PCNA, and Cdx2 protein expression (20x). (e–h) Semiquantitative analysis of cells expressing Musashi-1, TERT, PCNA, and Cdx2. Data are expressed as the mean ± SD (*n* = 5; ^∗∗^*P* < 0.005 and ^∗∗∗^*P* < 0.001 vs. saline group).

**Figure 6:**
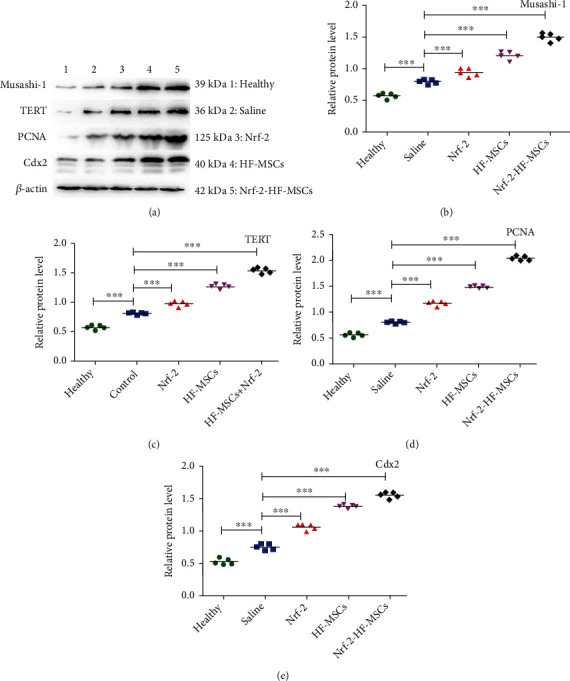
*Nrf-2* and HF-MSCs have important effects on ISC marker expression. (a) Western blot analysis of Musashi-1, TERT, PCNA, and Cdx2 protein expression in the different treatment groups. (b–e) Quantitative analysis of Musash-1, TERT, PCNA, and Cdx2 expression. *β*-Actin was used as a reference. Data are expressed as the mean ± SD (*n* = 5; ^∗∗∗^*P* < 0.001 vs. saline group).

IHC results revealed that marker expression in all five experimental groups tended to increase. Semiquantitative analysis was performed in three random high-power fields of view in each area (Figure 5). The WB results of Musashi-1, TERT, PCNA, and Cdx2 expression are shown in Figure 6. ISC marker expression was the lowest in the healthy group and increased in the order saline group < *Nrf-2* group < HF-MSC group < *Nrf-2*-HF-MSC group.

**Figure 7:**
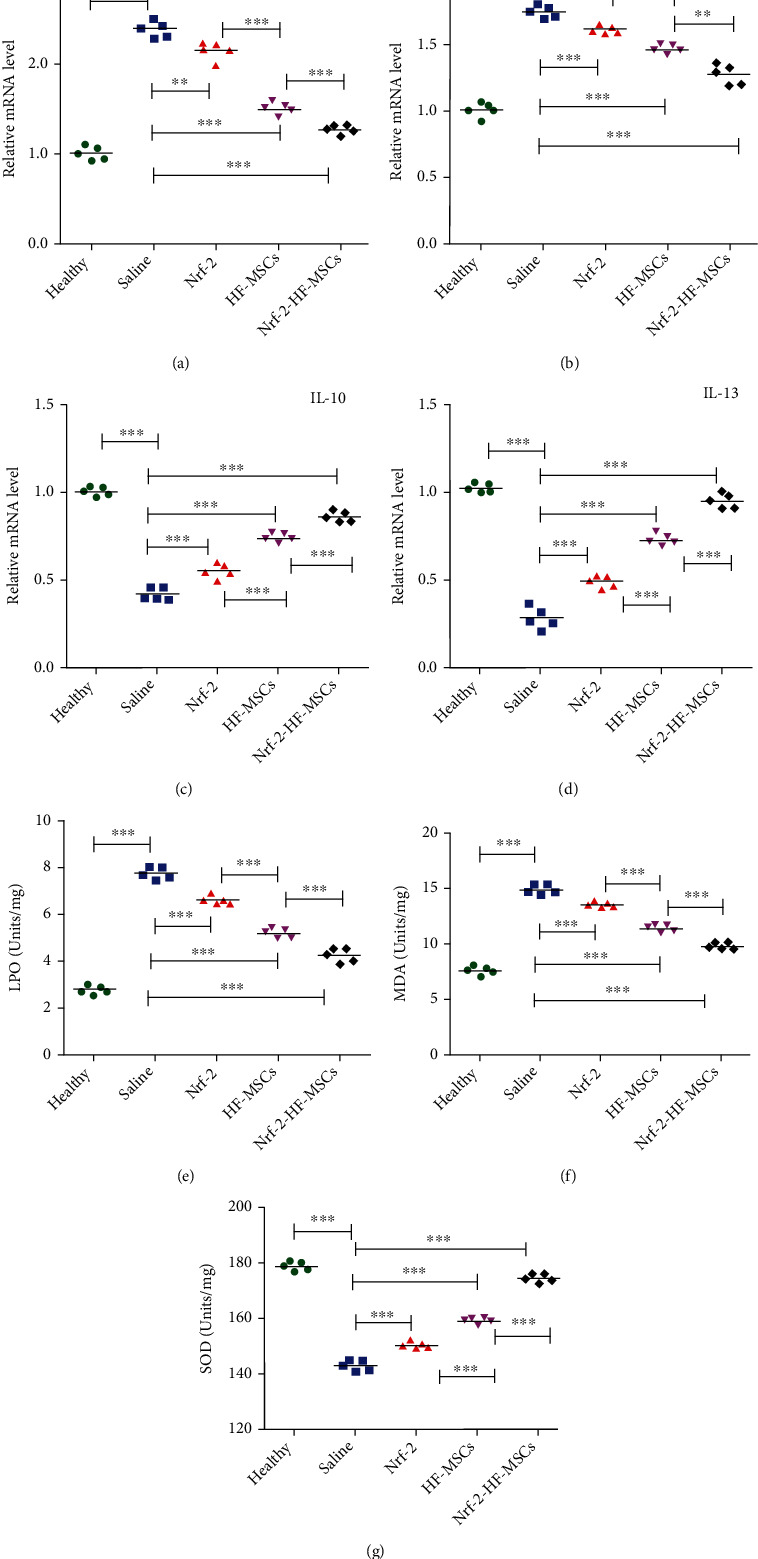
Significant effect of *Nrf-2* and HF-MSCs on inflammatory and anti-inflammatory factors and oxidative stress indices. (a–d) Quantitative analysis of *IL-1β*, *TNF-α*, *IL-10*, and *IL-13* mRNA expression by RT-qPCR. *β*-Actin was used as a reference. (e–g) Expression of LPO, MDA, and SOD in colon tissue. Data are expressed as the mean ± SD (*n* = 5; ^∗∗^*P* < 0.05 and ^∗∗∗^*P* < 0.001 vs. saline group).

RT-qPCR results indicated that inflammatory cytokines' mRNA expression, which includes IL-1*β* and TNF-*α*, was increased within UC rats' colon tissues. These increases were suppressed in the *Nrf-2*, HF-MSC, and *Nrf-2*-HF-MSC treatment groups. Rats in the *Nrf-2*-HF-MSC group exhibited the lowest expression of inflammatory factors (*P* < 0.05) (Figures 7(a) and 7(b)). In contrast, mRNA expression of the anti-inflammatory cytokines IL-13 and IL-10 was increased in colon tissues of rats treated with *Nrf-2*, HF-MSC, or *Nrf*-*2*-HF-MSCs when compared to the saline-treated UC rats (*P* < 0.05) (Figures 7(c) and 7(d)).

SOD, LPO, and MDA are the common indicators used to measure oxidative stress in cells. SOD has been regarded to be the antioxidant machinery's significant part within the biological systems. The SOD activity level indicates indirectly the competence of the body for scavenging oxygen free radicals. LPO and MDA are the products of lipoprotein peroxidation, which is a chain reaction producing free radicals from oxygen attacking the unsaturated fatty acids, resulting in extensive cell damage. This, in turn, damages the afflicted cells and organs. The assessment of LOP, MDA, and SOD levels indicated that HF-MSCs and *Nrf-2* alleviated the oxidative stress damage caused by UC (*P* < 0.05) (Figures 7(e) and 7(g)); the *Nrf-2* group was the least effective, followed by the HF-MSC group, while the *Nrf*-*2*-HF-MSC group was the most effective.

## 4. Discussion

This study revealed that *Nrf-2-*HF-MSCs promoted an increase in ISC, relieved disease symptoms, and reduced inflammation in UC model rats. Evidence suggests that MSCs mainly promote wound healing by (i) modulating the inflammatory response in wounds by regulating the functions of macrophages and T cells, (ii) producing vascular endothelial growth factor and basis fibroblast growth factor, (iii) promoting angiogenesis, (iv) promoting the proliferation and differentiation of fibroblasts and keratinocytes, and (v) neutralizing active oxygen on the wound surface [20–23]. In addition to their inflammatory response-regulatory activity, this finding may be due to the proliferation and differentiation of HF-MSCs into ISC or the secretion of certain substances to promote the growth of ISC.

There are numerous ISC markers, including Musashi-1, Cdx2, PCNA, and TERT. TERT is involved in the maintenance of telomere length, chromosomal stability, and cell activity, while promoting cell damage repair. In addition, TERT enhances cell proliferation *in vitro*. Musashi-1, a transcriptional repressor, maintains stem cell function by participating in Notch signaling pathway activation [24]. PCNA causes cell proliferation by binding to a cyclic protein kinase. PCNA expression can be used as an indicator of the cell proliferation status [25]. Cdx2 is a gut-specific transcription factor that is involved in the formation and differentiation of intestinal epithelial cells. Cdx2 is related to cell proliferation, adhesion, metastasis, and tumorigenesis [26, 27]. In theory, ISC would be the best source for intestinal repair and regeneration. Therefore, we used the above four intestinal stem cell markers in the IF, IHC, and WB analysis to assess the activity and proliferation of ISC in each experimental group.

Our results show that *Nrf-2* has a therapeutic effect on UC. Within the oxidative defense mechanism, a rather significant pathway is the Kelch sample related protein-1- (Keap1-) *Nrf-2*/antioxidant response element (ARE) signaling pathway, as it activates adaptive responses in cells to deal with various oxidative stress injuries [28, 29]. Since *Nrf-2* will dissociate from Keap1, the *Nrf-2* protein's level will increase within the cell under oxidative stress. Later on, *Nrf-2* will translocate to the nucleus, where it activates oxidative defense genes having the ARE sequence. Therefore, *Nrf-2* can induce the expression of a series of antioxidant genes, which is essential for preventing oxidative damage, inflammation, and tumorigenesis [30–34]. *Nrf-2*'s protective impacts on the colon were validated. *Nrf-2* protects intestinal integrity by regulating inflammatory cytokines and inducing phase II detoxifying enzymes. Within the colonic epithelium, multiple antioxidant systems are controlled by *Nrf-2*, which includes the antioxidant system based on glutathione, the Phase II drug conjugation system, hydrolysis system, reduction, and the Phase I drug oxidation [35]. Several *Nrf-2* activators have been proven to benefit colitis's treatment. For instance, sulforaphane pretreatment is able to mitigate acute colitis induced by DSS. Therefore, *Nrf-2* is a therapeutic target in UC. However, our experimental results clearly indicated that *Nrf-2* was not as effective as HF-MSCs and, certainly, *Nrf-2*-modified HF-MSCs.

The survival rate of MSCs in the recipient is not high. Studies have shown that pretreatment of MSCs can significantly increase their survival and differentiation rates and improve their damage repair ability [36, 37]. Such pretreatment serves to maintain the cells in an optimal state to adapt to *in vitro* stimulation and increase their resistance to oxidative stress injury after transplantation. Common methods include physical and chemical treatments, treatment with biologically active factors, and genetic modification. In this study, *Nrf-2-*HF-MSCs had a more obvious therapeutic effect than HF-MSCs and *Nrf-2*. In addition to the fact that *Nrf-2* and HF-MSCs exert their own effects on UC treatment, it is possible that *Nrf-2* enhances the therapeutic effects of HF-MSCs. This study had some shortcomings. UC also has extraintestinal manifestations, such as abnormal liver function, arthritis, and iridocyclitis, but we focused on intestinal symptoms only. Graft rejection, carcinogenicity, and other adverse effects did not occur during our study, but this may be due to an insufficiently long trial period.

In conclusion, *Nrf-2*-modified HF-MSCs recognized and migrated to UC lesions, repaired damaged intestinal tissue, inhibited the release of inflammatory factors while promoting that of anti-inflammatory factors, and thus cured DSS-induced ulcerative colitis in rats. According to the results, the transplantation of *Nrf-2* gene-modified rat-derived HF-MSCs is possible to be an affordable and useful therapeutic option for UC's clinical management.

## Data Availability

The data used to support the findings of this study are included within the article.
